# A new Gondwanan mayfly family from the Lower Cretaceous Crato Formation, Brazil (Ephemeroptera: Siphlonuroidea: Astraeopteridae fam. nov.)

**DOI:** 10.1038/s41598-023-36778-x

**Published:** 2023-07-20

**Authors:** Arianny P. Storari, Arnold H. Staniczek, Roman J. Godunko

**Affiliations:** 1 Laboratório de Paleontologia, Departamento de Ciências Biológicas, Centro de Ciências Humanas e Naturais, Universidade Federal do Espírito Santo, Vitória, Brazil; 2 Department of Entomology, State Museum of Natural History Stuttgart, Stuttgart, Germany; 3 Biology Centre of the Czech Academy of Sciences, Institute of Entomology, České Budějovice, Czech Republic; 4 Department of Invertebrate Zoology and Hydrobiology, University of Łódź, Łódź, Poland

**Keywords:** Palaeontology, Taxonomy, Entomology, Palaeontology, Evolution, Stratigraphy

## Abstract

The adult holotype of the fossil mayfly *Astraeoptera cretacica* Brandão et al. 2021 from the Cretaceous Crato Formation, Brazil, is reviewed and attributed to a new family Astraeopteridae fam. nov. Based on alate specimens, we also describe further new representatives of Astraeopteridae fam. nov., namely *Astraeoptera vitrea* sp. nov. and *Astraeoptera oligovenata* sp. nov., as well as the new genus and species *Eosophobia acuta* gen. et sp. nov. A subsequent character analysis of the new material suggests systematic affinities of Astraeopteridae fam. nov. with those extant families of Siphlonuroidea distributed in the Southern Hemisphere. These newly described fossil Siphlonuroidea from the Cretaceous of Brazil thus add to the biogeography and systematics of mayflies.

## Introduction

The Brazilian Araripe Basin with its Crato Formation represents one of the most important Lagerstätte for Cretaceous insects^1,2^. Particularly mayflies (Insecta, Ephemeroptera) are found most abundantly in these sedimentary limestones^3,4^. The dominant autochthonous part of the Crato mayfly fauna is represented by the species *Protoligoneuria limai* Demoulin, 1955 of the extinct family Hexagenitidae Lameere, 1917^5^. Nonetheless, nymphal and adult life stages of several other allochthonous groups can also be found. It is assumed that the nymphs were washed out from distantly connected lotic water bodies, while alate adults were reaching the depositional site through active flight^6,7^.

Among the allochthonous mayfly groups preserved in the limestones of the Crato Formation are Baetiscidae Leach, 1815; Ephemeridae Latreille, 1810; Euthyplociidae Edmunds & Traver, 1954; Oligoneuriidae Ulmer, 1914; Polymitarcyidae Banks, 1900; Potamanthidae Albarda, 1888; and Siphlonuridae Ulmer, 1920^3^. The latest addition to the knowledge on the Crato mayfly diversity was the description of a new genus and species *Astraeoptera cretacica* Brandão et al. 2021. However, Brandão et al.^8^ were not able to assign *Astraeoptera* to any fossil or extant higher group within Ephemeroptera, thus leaving their systematics in limbo.

In the present contribution, we review the adult holotype of *Astraeoptera cretacica*, evaluate its higher systematic affinities, and assign it to a new fossil family Astraeopteridae fam. nov. to be placed within the superfamily Siphlonuroidea Demoulin, 1958. We also describe new representatives of Astraeopteridae fam. nov., namely the two new species *Astraeoptera vitrea* sp. nov. and *Astraeoptera oligovenata* sp. nov., as well as the new genus and species *Eosophobia acuta* gen. et sp. nov.

## Results

Systematic Paleontology

Subphylum Hexapoda Latreille, 1825

Class Insecta Linnaeus, 1758

Order Ephemeroptera Hyatt & Arms, 1890

Superfamily Siphlonuroidea Demoulin, 1958

**Family** Astraeopteridae fam. nov.

urn:lsid:zoobank.org:act:5670D822-3A70-4C12-BB66-14ADF09DF170

**Figure 1:**
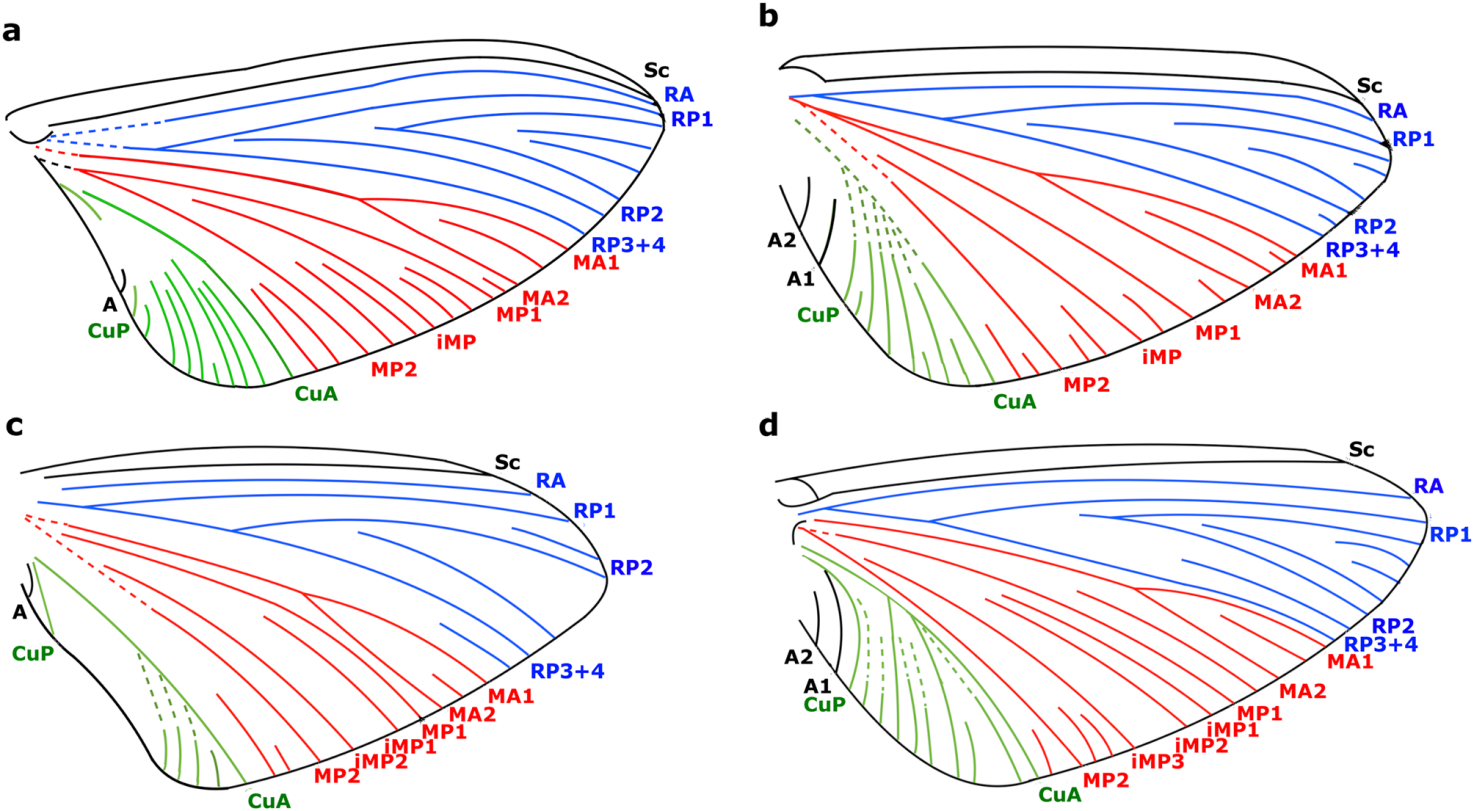
Forewing schemes with longitudinal veins of different Astraeopteridae fam. nov., cross veins are omitted. (**a**) *Astraeoptera cretacica* Brandão et al. 2021, LPRP/USP 0504. (**b**) *Astraeoptera vitrea* sp. nov., MPSC I 7437. (**c**) *Astraeoptera oligovenata* sp. nov., MPSC I 7438. (**d**) *Eosophobia acuta* gen. et sp. nov., MPSC I 7439.

**Figure 2:**
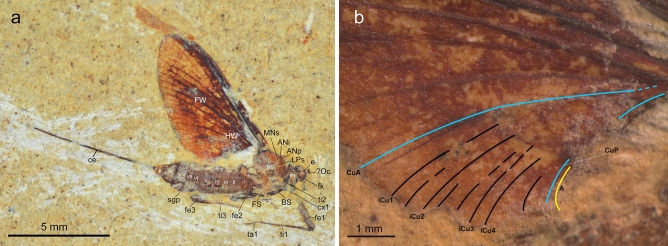
*Astraeoptera cretacica* Brandão et al. 2021, holotype, LPRP/USP 0504. Photographs adapted from Brandão et al. 2021. (**a**) General lateral view with reinterpretation of general body structures. Abbreviations: Head: *e* eye, *fk* facial keel, *Oc* frontal ocellus; Thorax: *ANi* anteronotal transverse impression, *ANp* anteronotal protuberance, *BS* basisternum, *FS* furcastemum, *FW* forewing, *HW* hind wing, *LPs* lateroparapsidal suture, *MNs* mesonotal suture, *MS* medioscutum, *PT* prothorax; Legs: *cx1* forecoxa, *fe1* forefemur, *fe2* middle femur, *fe3* hind femur, *ta1* foretarsus; *ta3* hindtarsus, *ti1* foretibia, *ti2* middle tibia, *ti3* hind tibia; Abdomen: *I* *X* abdominal segments, *ce* cercus, *sgp* subgenital plate. Scale bar 5 mm. (**b**). Interpretation of cubital field of forewing. Scale bar 1 mm.

**Type genus.** *Astraeoptera* Brandão et al. 2021 [by present designation] in^8^, 2, Figs. 1 and 2

**Generic composition.** Astraeopteridae fam. nov. include the two Cretaceous genera, *Astraeoptera* Brandão et al. 2021 and *Eosophobia* gen. nov.

**Locality and horizon.** Precise localities unknown, Northeast Brazil. Aptian, Lower Cretaceous, Crato Formation, Santana Group, Araripe Basin^9^.

**Diagnosis. Adult** (modified from^8^). Pterothorax more robust than in all other Siphlonuroidea, wider than prothorax, entire thorax markedly elevated above head in a straight line posteriad. Where preserved, mesonotal suture is more or less transverse (as in Siphluriscidae, Ameletopsidae, Nesameletidae, Oniscigastridae, and Rallidentidae); lateroparapsidal suture bent inward distally (as in all other families of Siphlonuroidea); furcasternal protuberances relatively large and apparently not contiguous (as in all other southern families of Siphlonuroidea). Cross veins present throughout entire forewing except of cubital field; MA branches in the apical half of wing at about 2/3 of MA length; MP2 and CuA straight at their bases; CuA field narrow, but larger than in other representatives of Siphlonuroidea; CuA simple, not forked; one–two intercalary veins between MP2 and CuA (typical of Siphlonuroidea but longer than in the extant representatives); cubital field with four–eight distinct intercalary veins, terminating at the outer margin of forewing (as in Oniscigastridae, Rallidentidae and Ameletopsidae). When preserved, the hind wing is well-developed, as long as 0.25–0.45 of forewing length (as in Ameletopsidae, Nesameletidae, Oniscigastridae, and Rallidentidae). Paracercus vestigial or markedly diminished.

**Comment.** It can be excluded that the markedly elevated thorax is an artifact of compression due to fossilization. The thorax is the most robust part of mayflies and hardly deformed during diagenesis and early stages of decomposition. If the thorax had been compressed, resulting in a change of shape, this would have been visible on the animal’s surface by cracks and shifting of thorax segments. In Crato mayflies, the thorax is either retained without deformation, or, when cracked, the thorax segments are shifted relative to each other. When subimaginal exuviae are preserved, the deformed thorax has a well-visible long dorsomedian opening (unpublished data from actualistic experiments and biostratinomic observations).

Genus ***Astraeoptera*** Brandão et al. 2021.

**Type species.** *Astraeoptera cretacica* Brandão et al. 2021 in^8^, 2, Figs. 1 and 2.

**Species composition.** *Astraeoptera cretacica* Brandão et al. 2021 [type species; adult female]; *Astraeoptera vitrea* sp. nov. [MPSC I 7437; adult male]; *Astraeoptera oligovenata* sp. nov. [MPSC I 7438; adult of putative female].

**Diagnosis. Adult.** Body length and forewing length ranging from 8.00 to 13.20 mm, and 7.65–11.40 mm, respectively. Thorax short, as long as approximately 0.5 of abdomen. Costal brace of forewing moderately arched; distal half of MA and MA1 not approximated to RP3 + 4; two intercalary veins between MP2 and CuA; cubital field with four–eight longitudinal veins subparallel to CuA, at least two–three of them nearly subparallel to CuA. Largest abdominal segments are VII and VIII.

*Astraeoptera cretacica* Brandão et al. 2021.

*Astraeoptera cretacica*: Brandão et al. 2021; *Cretaceous Research*, 127: 2, Figs. 1 and 2 [description; incomplete adult female (holotype: LPRP/USP 0504); Aptian, Lower Cretaceous, Crato Formation; within Euplectoptera *incertae sedis*].

**Emended diagnosis [based on adult female]** Body length 8.00 mm. Forewing length 7.60 mm; RP fork basally at 0.15 of forewing length; RP2 fork at about midlength of forewing; MA fork slightly asymmetrical; two long intercalaries between MP2 and CuA; cubital field with eight longitudinal veins subparallel to CuA. Hind wing as long as 0.25 of forewing length.

**Generalities.** Specimen preserved in lateral right view, with fragmented head and almost complete thorax and abdomen. Thorax damaged; mesothorax indistinct, especially mesonotum and shape of pleuron. Right forewing mostly complete except for anal field and cubital field; longitudinal venation distinguishable except for proximal part of RA and RP; base of MA, MP; and main cubital veins covered by matrix; cross veins partly poorly distinguishable. Hind wings present, but shape and venation not discernible. Fragments of [? right] foreleg, [? right] middle and hind legs, and fragment of [? left] hind leg preserved. One incomplete [? right] cercus preserved (Fig. 2a).

**Complementary description.** Adult female with body preserved in lateral aspect. Body length approximately 8.00 mm. Trace of head with elongated facial keel, at least as long as preserved part of putative eye. Scape short, poorly visualized; putative pedicel elongated and slim; only base of antennal flagellum preserved (Fig. 2a). For measurements see Supplementary Table 1.

Thorax in lateral view expanded above head. Prothorax narrow, at least 0.42 mm in length. Pterothorax well-developed, distally incomplete or partly lost, at least 2.40 mm long. Mesothorax large; mesonotum elongated; anteronotal protuberance [ANp] seems well developed and prominent; anteronotal transverse impression [ANi] well recognizable and relatively deep; putative remnants of mesonotal suture [MNs] stretched backward medially; distal end of putative remnants of lateroparapsidal suture [LPs] bent inwardly; traces of putative anterior paracoxal suture [PcxsA] elongated and seems to be complete; distal part of posterior arc of prealar bridge [PAB:PA] preserved with PAB:PA not shortened or reduced; furcasternum and basisternum relatively large (Fig. 2a).

Forewing relatively short and wide, length of preserved part 7.65 mm, width of preserved part 3.62 mm; ratio of wing length to width about 1.44. Costal brace poorly preserved at its distal end, apparently fused with costa and arched; costal field not widened at half-length, distally damaged, with at least 11 simple cross veins along 3/4 of wing length; distal part of pterostigma lost, with three or four forked cross veins in preserved part. Subcostal field between Sc and RA at half-length slightly narrower than costal field, with at least 17 simple cross veins frequently scattered distally; Sc and RA reaching wing apex. Solitary or paired intercalaries between RA and MA2; paired elongated free intercalaries between MP1 and CuA; additionally, a few short solitary veins between MA1 and cubital field. RP forked near base, after 0.15 of its length; two strong intercalaries in RP2 field; numerous simple cross veins between RA and RP3 + 4; at least 10 simple cross veins between RP2 and neighboring veins; RP2 proximally highly approximated to RP1; RP3 + 4 not branched. MA fork slightly asymmetrical, forked after 0.60 of its length; MA1 bent, MA2 nearly straight; MA1 and MA2 connected with iMA by four–six simple cross veins; MP fork slightly asymmetrical, forked basally after 0.13 of its length; with a pair of elongated, basally free intercalary veins between MP1 and iMP and also between iMP and MP2; basally iMP approximated to MP1, not shortened; MP2 straight at entire length; a pair of elongated, basally free intercalaries between MP2 and CuA, alternating with a few short intercalary veins. Cubital field relatively wide, with at least eight intercalary veins running probably from CuA towards posterior margin of wing; distal half of iCu1–iCu2 nearly subparallel to CuA, and putative ?iCu3–?iCu4 nearly subparallel to CuP; shorter free intercalaries alternating with iCu1–iCu4; CuP poorly visible; anal field not preserved, except of short fragment of putative [intercalary?] anal vein close to distal end of CuP (Fig. 2b). Hind wing fragmentarily preserved, incomplete, shape and character of venation not visible; preserved part of hind wing as long as approximately 0.37 of forewing (1.93 mm) (Fig. 2a).

Foreleg partly preserved; coxa as long as trochanter; forefemur as long as 0.5 of preserved part of foretibia; preserved part of foretarsus slightly shorter than foretibia; tarsomeres and pretarsal claw poorly discernible. Measurements for preserved parts of forelegs: femur—1.60 mm; tibia—3.00 mm; tarsus—1.55 mm. Middle legs shorter than foreleg, with partly preserved trochanters and relatively short femora. Hind femur partly preserved, with visible distal end; remnants of patellotibial suture poorly visualized on hind tibia basally; fragments of putative tarsus and pretarsal claw partly preserved; first tarsal segments of preserved legs not shortened (Fig. 2a).

Abdomen with relatively tall segments (possibly the result of fossilization); abdominal segments VII and VIII are the tallest and widest; remnants of relatively large subgenital plate posteriorly on sternum VIII; segment X smallest, details poorly visible. Only one terminal filament (either paracercus or cercus) partly preserved, fragmented, approximately 6.70 mm in length (Fig. 2a).

For a summary of morphological characters of *A. cretacica* compared to other representatives of Astraeopteridae fam. nov., see Supplementary Table 1.

*Astraeoptera vitrea* sp. nov.

urn:lsid:zoobank.org:act:4777BD3D-7C99-4548-A80F-59D0B73B8FDD

**Type material. Holotype.** Adult male, inventory number MPSC I 7437 (collection of Museu de Paleontologia Plácido Cidade Nuvens, Santana do Cariri, Brazil).

**Etymology.** Species epithet derived from the Latin adjective *vitreus* for glassy, as the preserved forewing of the holotype resembles shiny glass.

**Diagnosis.** Body length 10.40 mm. Forewing length 8.40 mm; costal brace arched and only slightly pronounced; fork of RP slightly distant from base, at 0.23 of forewing length; RP2 forked at 0.48 of forewing length; two intercalaries between MP2 and CuA (one long and one short); cubital field with seven longitudinal veins subparallel to CuA. Hind wing as long as 0.45 of forewing length.

**Figure 3:**
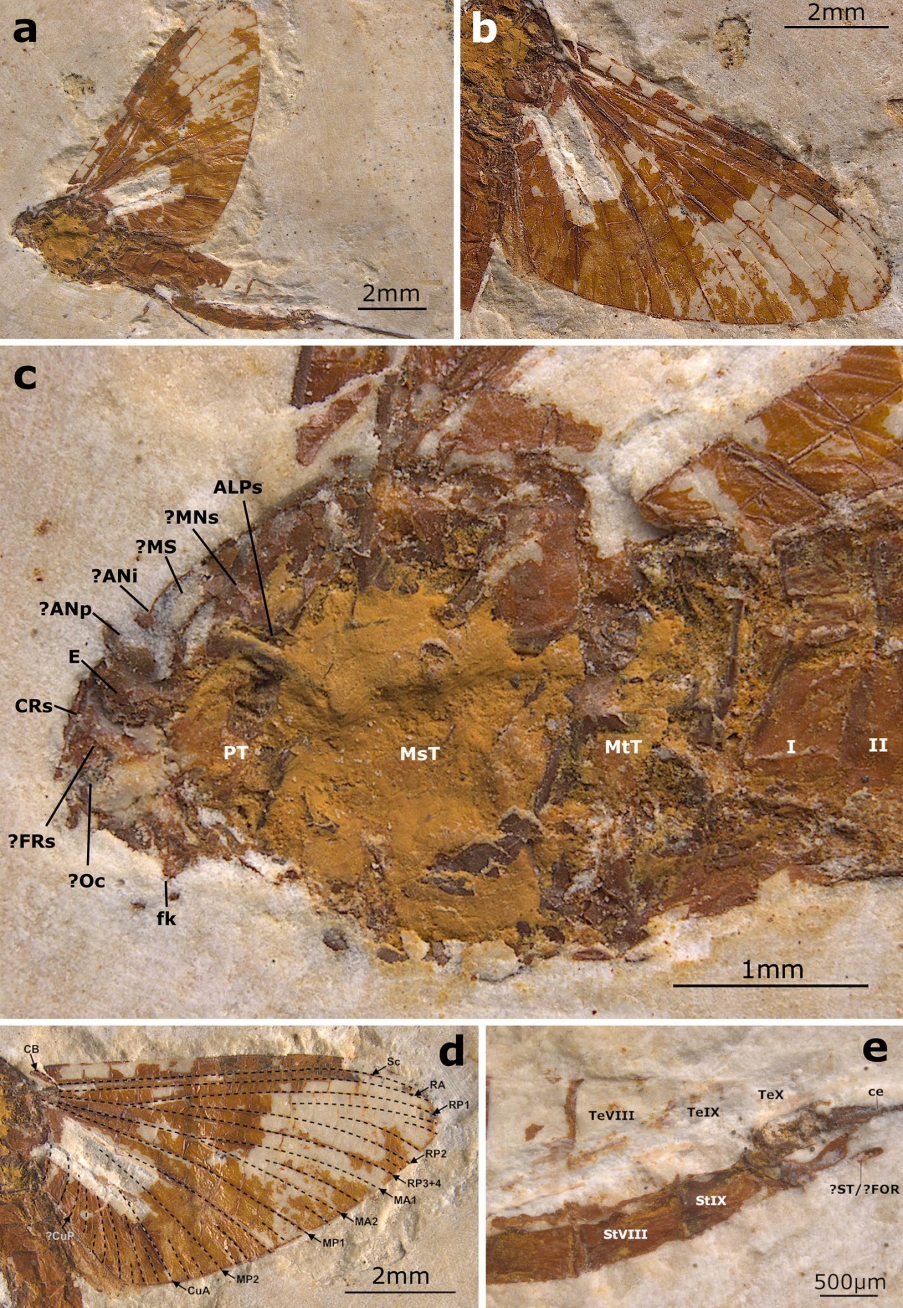
*Astraeoptera vitrea* sp. nov., holotype, adult male, MPSC I 7437. (**a**) General lateral view. Scale bar 2 mm. (**b**) Right forewing in dorsal view. Scale bar 2 mm. (**c**) Head, thorax and anterior abdomen in lateral view. Head: *E* eye, *CRs* coronal suture, *fk* facial keel; *FRs* frontal suture, *Oc* frontal ocellus; Thorax: *ANi* anteronotal transverse impression, *ALPs* antelateroparapsidal suture, *ANp* anteronotal protuberance, *MNs* mesonotal suture, *MS* medioscutum, *MsT* mesothorax, *MtT* metathorax, *PT* prothorax; Abdomen: *I and II* abdominal segments. Scale bar 1 mm. (**d**) Right forewing with an interpretative overlay of venation, *CB* costal brace. Scale bar 2 mm. (**e**) Posterior end of abdomen. *StVIII and StIX* abdominal sterna, *TeVIII–TeX* abdominal terga, *ce* base of cercus, *?ST/?FOR* styliger/forceps. Scale bar 500 µm.

**Generalities.** Specimen preserved in lateral left view, with partly preserved head, thorax and abdomen. Head and thorax damaged; details of mesopleuron not discernible; mesonotum poorly preserved; thoracic sterna mostly missing. Left forewing with almost completely preserved longitudinal and cross venation, except parts of iMP and MP2, and proximal half of cubital venation. Hind wings present, but shape and venation not discernible. Femur, tibia and part of putative tarsus of [? left] hind leg preserved; remnants [? femur and tibia] of putative [? right] hind leg partly preserved; remnants of putative middle leg preserved, separated from body. Abdominal terga V–X damaged or partly missing; genitalia damaged, incomplete. One incomplete terminal filament [? left cercus] preserved (Fig. 3a,b).

**Description.** Adult male with robust body embedded in lateral aspect. Body length approximately 10.4 mm. Head preserved but mostly damaged, with visible remnants of vertex, frons and clypeus; frontal and coronal sutures well distinguishable. Remnants of putative left eye mostly preserved, relatively small; right eye damaged; remnants of frontal ocellus relatively large. Antennae and ocelli missing. Facial keel relatively narrow (Fig. 3c). For measurements see Supplementary Table 1.

Thorax in lateral view expanded above head. Prothorax narrow, approximately 0.38 mm in length. Pterothorax short, as long as approximately 1/3 of abdomen (3.38 mm in length). Mesothorax large; mesonotum elongated; MNs poorly distinguishable (Fig. 3c).

Preserved left forewing elongated and wide; length of preserved part 8.45 mm, width of preserved part 4.53 mm; forewing width/length ratio—0.54. Longitudinal venation mostly well visible; cross venation moderately developed. Costal brace partly preserved basally, slightly pronounced, clearly arched; costal field between C and Sc narrow, with regular row of at least 17 simple cross veins, interspersed with about five forked cross veins in distal half; venation of pterostigma poorly preserved. Sc and RA straight at entire length; subcostal field narrower than costal field, with at least 13, sparsely scattered simple cross veins; Sc and RA reaching wing apex. Solitary intercalaries between RA and MA2 basally connected to main longitudinal veins by cross veins; between MP1 and CuA also solitary and paired intercalaries. RP fork slightly distant from base after 0.23 of RP length; RP2 forked near midlength. Fork of MA nearly symmetrical at about midlength (0.46); iMA is at least 0.57 of MA2 length; short solitary intercalary veins on each side of iMA. MP1–iMP–MP2 bent centrally; MP fork slightly asymmetrical; at least one short intercalary vein between MP1and iMP, and at least two of such intercalaries are between iMP and MP2; proximally iMP seems to be approximated to MP1, not shortened; MP2 straight at entire length; one elongated intercalary vein between MP2 and CuA, alternating with a pair of shorter intercalaries. Cubital field relatively narrow, with at least seven iCuA of different length, with iCu1–iCu4 nearly subparallel to CuA centrally and distally; putative CuP moderately bent distally. Anal field with one or two veins, moderately bent centrally. Hind wings preserved, but details of shape and venation are poorly distinguishable; preserved part of hind wing approximately 3.72 mm in length, as long as 0.44 of forewing (Fig. 3b,d).

Hind legs fragmentarily preserved, with indistinct margins between segments; distal end of [? left] hind leg reaches out to the middle of abdominal segment VI (Fig. 3a).

Abdomen relatively large, with moderately tall segments (possibly the result of fossilization); abdominal segment VII is the tallest and widest; remnants of putative male genitalia on segment X are present, but poorly preserved. Only one terminal filament [? left cercus] partly preserved, fragmented, with approximately 3.00 mm in length (Fig. 3a,e).

**Comments.** General similarities between *Astraeoptera vitrea* sp. nov. and *A. cretacica* (apart from familial and generic diagnostic characters) are the head wider than long; tornoapical margin of forewing about twice as long as basitornal margin; costal field not widened centrally; Sc and RA straight; subcostal field tapered centrally, narrower than costal field; RP basally branched at about 1/4 of wing length; MA2 nearly straight; iMA equidistant of MA1 and MA2; MP basally slightly asymmetrical branched with very short common stem; iMP slightly approximated to MP1; segment VII of abdomen is the widest (see Supplementary Table 1). For a summary of morphological characters of *A. vitrea* sp. nov. compared to other representatives of Astraeopteridae fam. nov., see Supplementary Table 1.

*Astraeoptera oligovenata* sp. nov.

urn:lsid:zoobank.org:act:DBB49369-612F-4F69-A0A0-FD877B08BCB5

**Type material. Holotype.** Adult [?] female, inventory number MPSC I 7438 (collection of Museu de Paleontologia Plácido Cidade Nuvens, Santana do Cariri, Brazil).

**Etymology.** Species epithet named after its fewer numbers of cubital intercalary veins compared to its congeners, from Latin *oligo* for few and *venatus* for veined.

**Diagnosis.** Body length 13.20 mm. Basisternum of mesothorax relatively short. Forewing length 11.40 mm; RP fork basally at 0.15 of RP length; RP2 fork basally at 0.26 of RP2 length; one intercalary between RP3 + 4 and MA; two intercalaries between MP2 and CuA (one short and one long); cubital field with at least three longitudinal veins subparallel to CuA.

**Figure 4:**
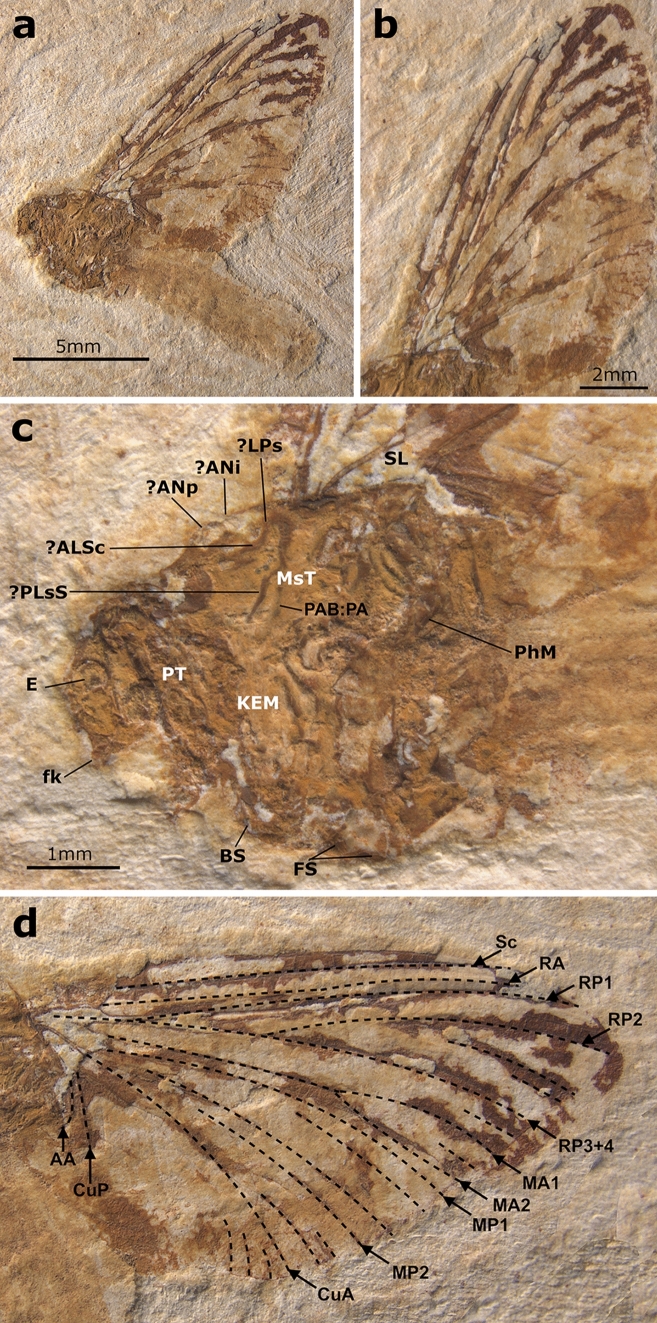
*Astraeoptera oligovenata* sp. nov., holotype, adult [?] female, MPSC I 7438. (**a**) General lateral view. Scale bar 5 mm. (**b**) Right forewing in dorsal view. Scale bar 2 mm. (**c**) Head and thorax in lateral view. Head: *E* eye, *fk* facial keel; Thorax: *?ALSc* anterolateral scutal costa, *?ANi* anteronotal transverse impression, *?ANp* anteronotal protuberance, *BS* basisternum, *FS* furcastemum, *KEM* katepimeron, *?LPs* lateroparapsidal suture, *MsT* mesothorax, *PhM* middle phragma, *?PLsS* superior pleural suture, *PAB:PA* posterior arc of prealar bridge, *PT* prothorax, *SL* scutellum. Scale bar 1 mm. (**d**) Right forewing with an interpretative overlay of venation. Scale bar 2 mm.

**Generalities.** Incomplete specimen embedded in left lateral position, with damaged head and thorax. Left forewing partly preserved; venation poorly distinguishable; hind wings and all legs missing; abdominal segments without details, only traces preserved; only margins of segments I–IV visible; caudal filaments missing (Fig. 4a,b).

**Description.** Adult, sex cannot be determined. Body length approximately 13.20 mm. Head fragmentarily preserved, eyes relatively large, at least 0.33 of head length; facial keel elongated and relatively wide proximally; antennal segments not preserved (Fig. 4a). For measurements, see Supplementary Table 1.

Thorax in lateral view expanded above head, poorly preserved. Prothorax relatively large, about 0.80 mm in length. Pterothorax well developed, robust, with poorly preserved pleuron and partly damaged metathorax; preserved part of pterothorax at least 5.00 mm in length. Mesothorax largest, with elongated mesonotum; structure and shape of MNs poorly visible, apparently stretched backward medially; putative LPs seems to be elongated and curved inward distally; PcxsA and PLsS well developed, elongated and complete; posterior arc of prealar bridge [PAB:PA] neither shortened nor reduced. Lateral and ventral aspect of thorax poorly visible; basisternum relatively short; putative furcasternal protuberance with indistinct shape and structure (Fig. 4c).

Forewing short and wide, length of preserved part 11.40 mm, width of preserved part at least 6.30 mm; width/length ratio of forewing is 0.55. Longitudinal and cross venation poorly preserved; cubital venation mostly missing; only distal ends of several cubital intercalaries preserved. Costal brace covered by matrix and not visible; costal field between C and Sc moderately widened centrally, lost distally; pterostigmatic area mostly missing; in preserved part of costal area at least six simple cross veins; Sc and RA nearly subparallel proximally, moderately bent distally; subcostal field centrally slightly wider than costal field. Sc reaching wing apex; RA terminating close to RP1. Intercalary veins between RP1 and CuA poorly visible and fragmented; their cross venation mostly missing; solitary or paired intercalaries preserved between RP1 and CuA. RP symmetrically forked after 0.15 of its length; RP2 slightly asymmetrical, forked after 0.26 of its length. MA nearly symmetrically forked at 0.58 of its length; MA1 moderately bent centrally and basally, MA2 slightly bent centrally; iMA poorly distinguishable, with preserved part of less than 1/3 of MA2 length; cross venation not visible. MP fork is covered by matrix; MP1 and MP2 moderately bent centrally; proximal part of MP2 missing; at least one elongated intercalary vein between MP1 and iMP, no visible intercalaries between iMP and MP2; cross venation of MP field mostly missing; two short intercalary veins between MP2 and CuA. Cubital field relatively narrow, with at least three veins running from CuA towards posterior margin of wing [the length of these veins cannot be determined]; remaining cubital field damaged; CuP fragmentary, preserved part straight. Anal field damaged, with a single preserved vein A1 clearly bent distally. Outline of one hind wing fragmentarily preserved, incomplete, at least 1/3 of forewing length; shape and structure of hind wing venation not visible (Fig. 4b,d).

All legs are missing. Abdominal segments I–IV nearly subequal in length. Caudal filaments missing (Fig. 4a).

**Comments.** The general similarities between *Astraeoptera oligovenata* sp. nov. and *A. cretacica* (apart from diagnostic characters of Astraeopteridae fam. nov. and *Astraeoptera*) are the positions of RP and MA forks and the shape of vein A1, which are strongly bent distally. General similarities between *A. oligovenata* sp. nov. and *A. vitrea* sp. nov. concern the forewing width/length ratio; the nearly symmetrical fork of MA; and the presence of two intercalaries between MP2 and CuA, one being short and one long (see Supplementary Table 1). Also important to note is that the cubital field of specimen SMF VI 743 is not entirely preserved, so the number of longitudinal veins of the cubital field is probably higher than only the three discernible ones. For a summary of morphological characters of *A. oligovenata* sp. nov. compared to other representatives of Astraeopteridae fam. nov., see Supplementary Table 1.

*Eosophobia* gen. nov.

Urn:lsid:zoobank.org:act:F1CC23ED-862E-43E2-9FB1-89997BF00969

**Etymology.** The generic name *Eosophobia* gen. nov. is a compound noun of female gender, deriving from Eos (Greek: Ἠώς), the Greek goddess of dawn, and phobia (Greek: φοβία), the Greek word for fear. Eos was the wife of titan Astraeus, the Greek god of dusk, after whom the related mayfly genus *Astraeoptera* was named. Eosophobia is also a common medical term describing the fear of dawn or daylight. It is also the name of a song by American musician Jack White III on the record “Fear of Dawn”, released in 2022, which was in heavy rotation when describing this genus.

**Type species.** *Eosophobia acuta* sp. nov.

**Species composition.** Monospecific.

**Diagnosis.** Body length 7.60 mm; forewing length 7.70 mm. Thorax elongated, as long as 0.82 of abdomen. Costal brace strongly arched, more than in *Astraeoptera*; relatively fewer cross veins than in *Astraeoptera*; RP fork at 0.22 of its length; RP2 fork at 0.20 of its length; distal half of MA + MA1 strongly approximated to RP3 + 4 (at least four times closer than in *Astraeoptera*); three long intercalaries between MP1 and MP2; one short intercalary between MP2 and CuA, instead of two as in *Astraeoptera*; cubital field with at least eight longitudinal veins in an acute angle of CuA, not subparallel as in *Astraeoptera*, only one vein nearly subparallel to CuA. Largest abdominal segments of abdomen are V and VI (unlike VII and VIII in *Astraeoptera*).

*Eosophobia acuta* sp. nov.

urn:lsid:zoobank.org:act:F3200004-006F-4917-8BC9-4BB64D33EA6A

**Type material. Holotype.** Adult female, inventory number MPSC I 7439 (collection of Museu de Paleontologia Plácido Cidade Nuvens, Santana do Cariri, Brazil).

**Etymology.** The specific epithet is of female gender and derived from the Latin adjective *acutus* (sharp), referring to the posterior veins of the cubital field of forewing, which are arranged in an acute angle to CuA, compared to the respective subparallel orientation of these veins in the cubital fields of its congeners. At the same time, *acuta* refers to an acute condition of eosophobia.

**Diagnosis.** As for *Eosophobia* gen. nov., as monospecific.

**Figure 5:**
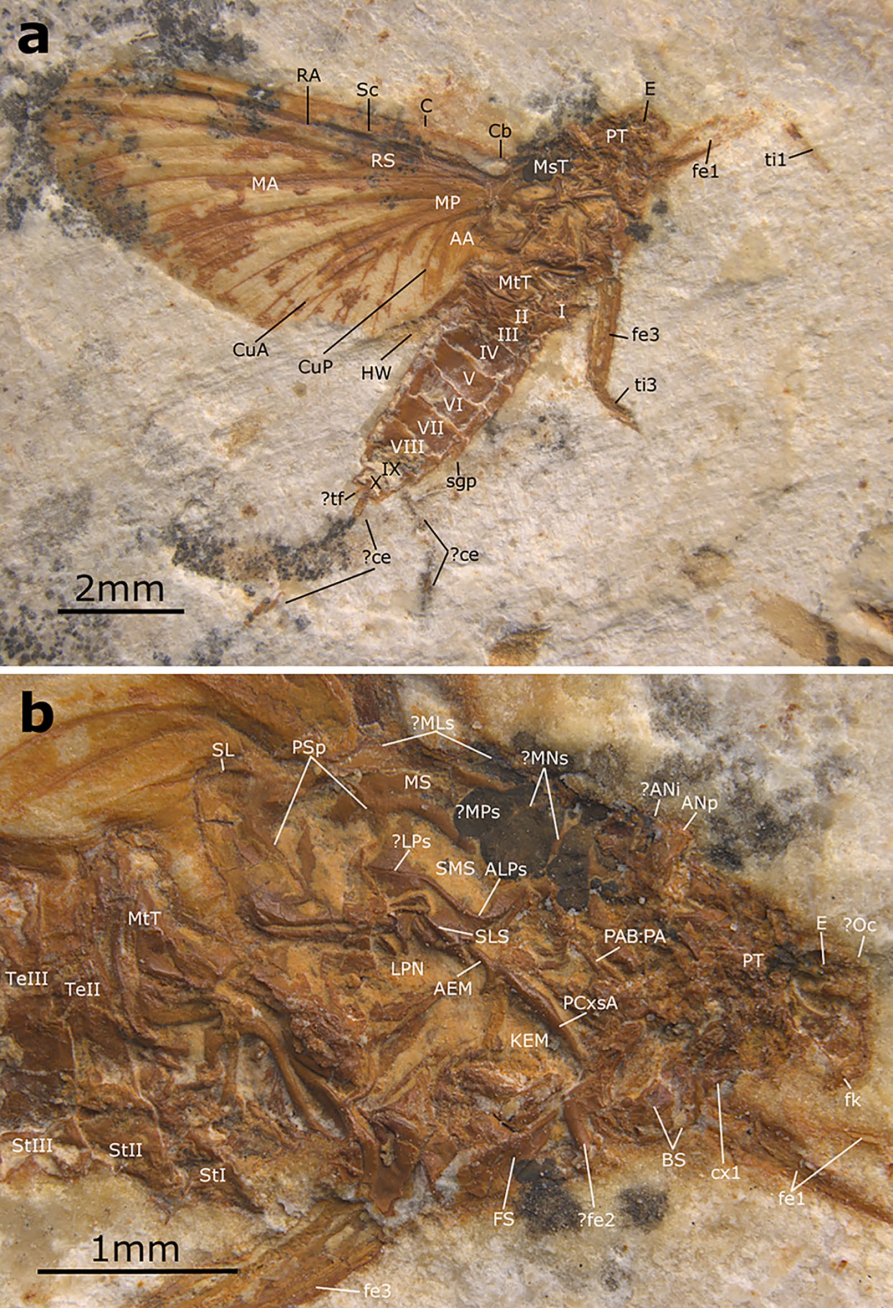
*Eosophobia acuta* sp. nov., holotype, adult female, MPSC I 7439. (**a**) General view in lateral attitude with reinterpretation of general body structures. Abbreviations: Head: *E* eye; Thorax: *MsT* mesothorax, *MtT* metathorax, *PT* prothorax; Legs: *fe1* forefemur, *fe3* hind femur, *ti1* foretibia, *ti3* hind tibia; Wings: *AA* anal anterior, *C* costa, *Cb* costal brace, *CuA* cubitus anterior, *CuP* cubitus posterior, *HW* hind wing, *MA* media anterior, *MP* media posterior, *RA* radius anterior, *RS* radius sector, *Sc* subcosta; Abdomen: *I–X* abdominal sterna segments, *ce* cercus, *sgp* subgenital plate, *tf* terminal filament. Scale bar 2 mm. (**b**) Head and thorax in lateral view. Head: *E* eye; *fk* facial keel, *Oc* frontal ocellus; Thorax: *AEM* anepimeron, *ALPs* antelateroparapsidal suture, *ANi* anteronotal transverse impression, *ANp* anteronotal protuberance, *BS* basisternum, *FS* furcastemum, *KEM* katepimeron, *LPN* lateropostnoturn, *LPs* lateroparapsidal suture, *?MLs* median longitudinal suture, *MNs* mesonotal suture, *MPs* medioparapsidal suture, *MS* medioscutum, *MtT* metathorax, *PCxsA* anterior paracoxal suture of mesothorax, *PAB:PA* posterior arc of prealar bridge, *PSp* posterior scutaI protuberance, *PT* prothorax, *SL* scutellum, *SLS* sublateroscutum, *SMS* submedioscuturn; Legs: *cx1* forecoxa, *fe1* forefemur, *fe2* middle femur, *fe3* hind femur; Abdomen: *StI–III* abdominal sterna, *TeII–III* abdominal terga. Scale bar 1 mm.

**Figure 6:**
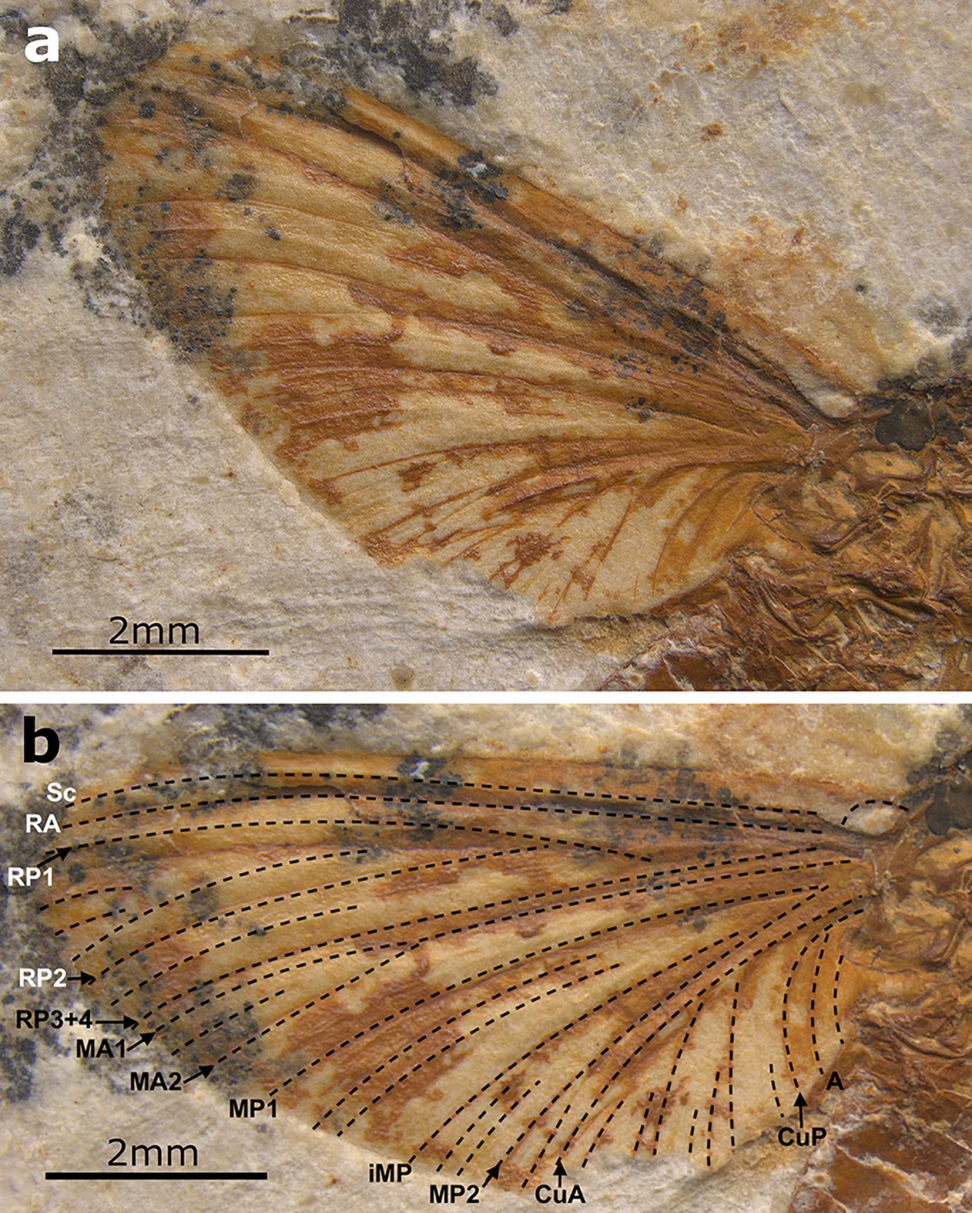
*Eosophobia acuta* sp. nov., holotype, adult female, MPSC I 7439. (**a**) Right forewing in ventral view. Scale bar 2 mm. (**b**) Right forewing in ventral view with an interpretative layer of venation. Scale bar 2 mm.

**Generalities.** Well-preserved specimen visible in lateral view, with an almost complete body. Head with preserved eye and putative frontal ocellus. Thorax almost complete, with pleuron relatively well visible. Longitudinal venation relatively well preserved; pattern of forewing venation hardly distinguishable due to overlapping of veins in both forewings. Distal end of preserved right forewing partly damaged or lost. Hind wing preserved, but detailed shape and venation pattern not discernible. Right fore- and hind legs fragmentarily preserved; tarsi and pretarsal claws missing. Abdominal segments completely preserved, with putative subgenital plate ventrally on segment VII partly damaged; abdominal segments IX–X damaged, subanal plate not discernible; traces of both putative cerci preserved; putative paracercus probably vestigial (Figs. 5 and 6).

**Description.** Adult of putative female with body well visible in lateral view. Body length approximately 7.6 mm. Head mostly damaged; preserved trace of eye relatively large, at least 0.40× of head length; relatively wide trace of frontal ocellus. Antennae and ocelli missing. Facial keel slightly elongated, distally hooked, pointed, and strongly bent inwardly (Fig. 5a). For measurements see Supplementary Table 1.

Thorax in lateral view expanded above head. Prothorax narrow, approximately 0.47 mm in length. Pterothorax robust, distinctly elongated, 0.82× of abdomen length (3.50 mm in length). Mesothorax large; mesonotum elongated, with MNs stretched backward medially; putative medioparapsidal suture [MPs] and lateroparapsidal suture [LPs] concave centrally, distally bent inwards. Anterior paracoxal suture [PcxsA] well developed and complete; posterior arc of prealar bridge [PAB:PA] neither shortened nor reduced. Ventral aspect of thorax not visible; putative furcasternal protuberance partly visible in lateral view, relatively large, tapering anteriorly, apparently not contiguous; basisternum not large. Mesothorax and metathorax with preserved scutellum (Fig. 5b).

Preserved right forewing relatively narrow; tip of forewing damaged; length of preserved part 7.7 mm, width of preserved part 3.7 mm. Longitudinal venation mostly well recognizable; cross venation moderately developed, poorly visible. Cubital brace well preserved, strongly arched; costal area narrow, with relatively dense row of at least 13 simple cross veins proximally and centrally; sparse, forked veins spread distally, including pterostigma; details of pterostigma venation poorly recognizable. Sc and RA straight at entire length and nearly subparallel proximally and centrally, divergent distally, reaching wing apex; subcostal area relatively narrow; cross venation poorly preserved, at least eight simple cross veins hardly distinguishable. RP nearly symmetrical, fork clearly distant from base; RP forked after 0.22 of its length; RP2 nearly symmetrical, forked at about 0.20 of its length. RP3 + 4 approximated to MA1 distally. Several long and short solitary intercalaries between RP and CuA. Fork of MA nearly symmetrical, situated distally, forked after 0.65 of its length; iMA as long as 0.66 of MA2 length. MP1–iMP–MP2 slightly bent distally; MP fork seems to be asymmetrical and situated close to vein base; MP forked at least after 0.07 of its length; iMP not shortened, approximated MP2 basally; paired intercalary veins on each side of iMP; MP2 and CuA not convergent basally, straight at entire length; one short intercalary vein between MP2 and CuA. Cubital field relatively narrow, with at least seven iCuA of different length running from CuA towards posterior margin of wing, terminating at basitornal margin; iCu3 and iCu6 longest; cross venation in cubital field poorly preserved; CuP distinctly bent. Anal field with at least two moderately bent veins; A1 basally approximating CuP (Fig. 6). Hind wings preserved, but details of shape and venation poorly distinguishable; preserved part of hind wing as long as approximately 0.44 of forewing; traces of hind right wing of 3.40 mm in length.

Right foreleg fragmentarily preserved, with relatively wide femur; preserved part of tibia longer than femur. Preserved part of hind right femur relatively wide centrally; longer tibia (Fig. 5a).

Abdomen relatively short and robust, segments moderately tall (possibly the result of fossilization); abdominal segments V–VIIII approximately of same width, segments V–VI largest; segment X partly damaged, subanal plate missing; structure of putative subgenital plate indistinct, its distal half lost. Fragmented remnants of putative cerci shorter than body; putative remnant of non-segmented paracercus terminates at abdominal segment X (Fig. 5a).

## Discussion

When Brandão et al.^8^ described *Astraeoptera cretacica* as a new genus and species, they refrained from placing it in an existing or new mayfly family, stating that the specimen lacked crucial characters for a proper assignment and thus leaving its systematic position in limbo.

Now, with several additional specimens available, which could be placed in four different species and two genera, it is possible to narrow down the systematic affinities of these mayflies.

Several characters clearly indicate that the new taxon belongs to the crown-group Ephemeroptera. All specimens have a significantly reduced size of hind wing, whereas the forewing shows the characteristic, apomorphic type of costal brace, which is basally fused with the costal vein (unlike the unfused condition as seen in Paleozoic and Mesozoic Ephemerida, see^10^).

Within the Ephemeroptera, Astraeopteridae fam. nov. clearly form a monophyletic group. Unlike all other mayflies, adult Astraeopteridae fam. nov. have an autapomorphic, robust thorax. Unlike the humped pterothorax in all other mayflies, the entire thorax of Astraeopteridae fam. nov. is markedly ascending above the head in a straight angle posteriad, giving the thorax a unique appearance in lateral view (Figs. 2, 3, 4 and 5).

Otherwise, most of the characters visible are plesiomorphic within Ephemeroptera, so it is on the one hand difficult to find synapomorphic characters, which would place Astraeopteridae fam. nov. as sister group to another monophyletic group within Ephemeroptera. On the other hand, it is actually easy to preclude their systematic placement within several of the traditional superfamilies or other higher taxa (see below).

The anteritornous wing shape precludes a placement within Prosopistomatoidea. The first tarsomere, which is not shortened in the imaginal stage (preserved in the type species *A. cretacica*, Fig. 2a), precludes a placement within the superfamilies Ephemerelloidea, Caenoidea, Leptophlebioidea, and Ephemeroidea (all united as Furcatergalia sensu Kluge^11^). Likewise, Astraeopteridae fam. nov. cannot be placed within Heptagenioidea (i.e. Branchitergalia sensu Kluge^11^), or Setisura (sensu McCafferty^12^) due to their well-developed posterior arc of the prealar bridge (PAB:PA, see Figs. 2a, 4c and 5b), which is neither shortened nor reduced in both genera like it is in the Heptagenioidea. Finally, Astraeopteridae fam. nov. probably have also no closer relationship with Baetoidea. While it is not possible to evaluate the numbers of tarsomeres in the legs of the two genera of Astraeopteridae fam. nov. due to their poor preservation, the combination of a well-developed transverse mesonotal suture and the basal approximation of CuA to CuP in the forewing still precludes the inclusion of Astraeopteridae fam. nov. into Baetoidea.

While Astraeopteridae fam. nov. obviously cannot be placed within most of the superfamilies of Ephemeroptera as discussed above, their forewings resemble in many ways the plesiomorphic forewings present in Siphlonuroidea, with whom they have in common the characteristic cubital field containing a series of sinusoid, long, intercalary veins, occasionally forked towards the hind margin of the forewing. Together with the typical, probably plesiomorphic swimming setation of the tail filaments in the nymphs as already seen in Mesozoic Protereismatidae, this common plesiomorphic wing feature together with the lack of other apomorphic characters has been regarded as the main defining character for Siphlonuroidea^13^. It is obvious that Siphlonuroidea are an artificial, paraphyletic assemblage of different lineages, which only share symplesiomorphic characters, also molecular analyses did not support Siphlonuroidea as monophyletic^14,15^. However, Astraeopteridae fam. nov. on the one hand is a distinct phylogenetic lineage with autapomorphic characters, namely the unique shape of the thorax. On the other hand, it is lacking any obvious synapomorphic characters to place it within any other superfamily. Consequently, we place the new taxon provisionally as a separate family within Siphlonuroidea, knowing that the lack of many key characters, used in Ephemeroptera systematics, on the few specimens of Astraeopteridae fam. nov. precludes a thorough cladistic analysis at this time.

Kluge et al.^13^ distinguished within Siphlonuroidea a group of families distributed in the Northern Hemisphere and another group in the Southern Hemisphere. In the Northern Hemisphere group, the mesofurcasternal protuberances are contiguous over their entire length, while in the Southern Hemisphere group, the mesofurcasternal protuberances are separated by a median invagination at least in their posterior part to give space to the metathoracic ganglion, which has shifted anteriorly in these taxa. However, the enigmatic family Siphluriscidae, only known from the single species *Siphluriscus chinensis* Ulmer, 1920, was not considered by Kluge et al.^13^, although it likewise fulfills the formal criteria for placement within Siphlonuroidea. While distributed in the Northern Hemisphere, namely in Southern China and Vietnam^16^, it still shows separated mesofurcasternal protruberances, at least in their posterior parts. It is difficult to evaluate this character in Astraeopteridae fam. nov., because all species described here are preserved in lateral position and only a small part of the thoracic sterna is recognizable. Only in *Eosophobia* gen. nov. we can confirm the presence of a relatively large mesofurcasternal protuberance, which is tapering anteriorly and apparently not contiguous, as in Siphluriscidae Zhou and Peters, 2003 and the siphlonuroid families of the Southern Hemisphere^11,13^. However, this character in any case is of limited phylogenetic significance, as an anteriorad shift of the metathoracic ganglion has taken place several times in different groups of Ephemeroptera^11^.

For a broad taxonomic and paleoecological comprehension of Siphlonuroidea placement in the Cretaceous of Brazil, it is also paramount to re-evaluate McCafferty’s^17^ unnamed putative records of adult Siphlonuridae. Based on the descriptions and illustrations provided, it is difficult to judge the precise systematic position of the material. It is only certain that all specimens are located within Anteritorna. However, at least the alate specimen preserved laterally and depicted in his figure 13 [AMNH 43477] does not possess the autapomorphic markedly elevated thorax. The head and thorax of two other putative adult siphlonurids discussed by McCafferty^17^ are either missing [AMNH 44313] or very damaged [AMNH 44306]. Finally, the base of most veins of the forewing, including MP2 and CuA, appears to be poorly preserved or covered by the matrix in all discussed specimens. Therefore, it is difficult to establish the presence/absence of bending of MP2 and CuA veins posteriorly at the base. McCafferty^17^ already even discussed that siphlonurids presence in Brazil was predictable since the group crossed the equator at least twice and probably through West Gondwana.

Apart from Astraeopteridae fam. nov. found in the Cretaceous of Brazil, only a few Gondwanan representatives of Siphlonuroidea of the Southern Hemisphere have been reported from the Cretaceous of Australia^18^. On the contrary, there is a remarkable diversity of fossil Siphlonuroidea concentrated in Eurasian deposits, which include Germany (Jurassic), France (Triassic), Ukraine (Triassic), China (Jurassic), Mongolia (Jurassic and Cretaceous), Russia (Jurassic, Cretaceous, and Neogene), Poland (Paleogene), and Japan (Neogene)^19–31^. For a complete list of fossil records of Siphlonuroidea see Supplementary Table 2. This may represent a collector bias, but more likely it reflects a biogeographic center of many basal lineages in the Northern Hemisphere^32^, with only a few extant representatives in four small families, mostly of amphinotic distribution in the Southern Hemisphere, namely Oniscigastridae, Ameletopsidae, Nesameletidae, and Rallidentidae.

Fossil Gondwanan representatives of Siphlonuroidea are the bottom-dwelling larvae *Australurus plexus* Jell and Duncan, 1986; *Dulcimanna sculptor* Jell and Duncan, 1986; and *Promirara cephalota* Jell and Duncan, 1986, which were all described as Siphlonuridae. *A. plexus* is considered a common species and part of the autochthonous fauna of the Koonwarra beds, just as the hexagenitid larvae *Protoligoneuria limai* is considered for the Crato Formation^4^. However, the Koonwarra paleolake, unlike Crato, is hypothesized as a shallow, cold, and freshwater water setting^18^. In the Crato Formation, Astraeopteridae fam. nov. may have prospered in opening phases of periodic floodings in larger distant depressed areas, as already assumed as more humid phases of the Crato system^33^.

Siphlonuroidea, despite their nymphs having a morphotype typical of lentic settings, do not usually inhabit equatorial regions but are distributed in more temperate climates^17,32^. It is important to point to the hypothesized arid paleoclimate of the Crato Formation in its main member Nova Olinda^33^, and also the physical features of most of the water body phases in the deposition paleolake itself (closed system)^34,35^. The former was probably favourable for the Hexagenitidae, as evidenced by an autochthony of this group^4,5^, but harsh for other groups such as the Oligoneuriidae^36^, Euthyplociidae^6^, and most certainly also Astraeopteridae fam. nov. These, as part of a significant allochthonous fauna, apart from being rare in this deposit, are more commonly found as adults that must have reached the depositional site through flight, and their rarer larvae washed out during wetter and open periods^6^. Here, we hypothesize powered flight for Astraeopteridae fam. nov., since they present a robust thorax with putative strong flight muscles, as well as wings with strong, complete venation in the radial and cubital field, which may have been also important for dispersal ability.

## Material and methods

The holotype of *Astraeoptera cretacica* is housed in the fossil collection of the Laboratório de Paleontologia de Ribeirão Preto, Universidade de São Paulo, Ribeirão Preto, Brazil under the accession no. LPRP/USP 0504, but was physically not accessible to us, so we relied solely on the documentation of Brandão et al.^8^ for our redescription. The remaining material was studied between 2016 and 2019 as a part of the fossil collection of Senckenberg Naturmuseum, Frankfurt/Main, Germany [SMF], who had acquired it in 2004 from Fossils Worldwide, Sulzbachtal, Germany, and later kindly made it available to us for investigation. Due to an exchange of specimens under the framework of an ongoing cooperation of the Senckenberg Gesellschaft für Naturforschung, Frankfurt/Main, Germany (SMF), with the Universidade Regional do Cariri (URCA), Crato, Brazil, the following holotypes are now curated at the Museu de Paleontologia Plácido Cidade Nuvens, Santana do Cariri, Brazil (MPSC): *Astraeoptera vitrea* Storari, Staniczek & Godunko, 2023 (former inventory number SMF VI 1026) has received inventory number MPSC I 7437, the holotype of *Astraeoptera oligovenata* Storari, Staniczek & Godunko, 2023 (inventory number SMF VI 743) has received the inventory number MPSC I 7438, and the holotype of *Eosophobia acuta* Storari, Staniczek & Godunko, 2023 (inventory number SMF VI 802) has received the inventory number MPSC I 7439.

The material was examined in dry condition, as well as under a layer of ethanol using stereomicroscopes Olympus SZX7 and Leica M205 C. Photographs were taken through a Leica Z16 APO Macroscope equipped with a Leica DFC450 Digital Camera using Leica Application Suite v. 3.1.8. Resulting photo stacks were processed with Helicon Focus Pro 6.4.1 to obtain combined photographs with extended depth of field. Photographs were sharpened, and contrast and tonality were adjusted using Adobe Photoshop™ version 23.1.1 (Adobe Systems Incorporated, San Jose, USA). All drawings were made using a Wacom tablet and the software Autodesk Version 8.6.1.

Measurements were taken either by using an ocular grid or inferred from the photographs taken with a calibration scale (see Supplementary Table 1).

The descriptive morphological terminology mainly follows Kluge^11,37^, for the systematic classification we followed Kluge^11,13^.

## Conclusion

A new extinct mayfly family, Astraeopteridae fam. nov., is described from the Lower Cretaceous Crato Formation. This new taxon comprises two genera, the genus *Astraeoptera*^8^, and the monospecific genus *Eosophobia* gen. nov., assigned in the present study. The type genus of the new family, *Astraeoptera*, is composed of three species, the type species *Astraeoptera cretacica* Brandão et al., 2021, and the two newly described *Astraeoptera vitrea* sp. nov., and *Astraeoptera oligovenata* sp. nov. After comparison and addition of new specimens to the taxon, we place Astraeopteridae fam. nov. within the basal, putatively paraphyletic Siphlonuroidea. So far, only adult forms were described for the group, but even though Astraeopteridae fam. nov. were most certainly living at distance to the depositional site of the Crato, it might not be impossible to discover their immatures after investigation of many unstudied larvae in numerous collections or in new material from new excavations at this Lagerstätte. A thorough review that includes first-hand investigation of putative Siphlonuridae specimens described by McCafferty^17^ is also paramount to clarify the occurrence of further Siphlonuroidea among the Crato mayfly fauna and broadly in the Gondwana, as well as the possibility of association of these specimens within Astraeopteridae fam. nov.

### Supplementary Information

Supplementary Table 1.

Supplementary Table 2.

## Data Availability

All data generated or analysed during this study are included in this published article and its Supplementary Information files. All relevant data are available from the authors. The datasets generated during and/or analysed during the current study are available from the corresponding author upon reasonable request. All fossil specimens newly described in this study are housed in the institutional collection of Museu de Paleontologia Plácido Cidade Nuvens, Santana do Cariri, Brazil (MPSC), as specified in the Section “Material and methods”. Respective inventory numbers of studied specimens are listed in this published article. Requests for access to the fossil materials should be addressed to the curator of the collection. This work has been registered online at zoobank.org under LSID urn:lsid:zoobank.org:pub:1A5D1BE4-2B49-4D36-8745-9D9A1A700D92. The new taxa are registered in zoobank.org.
