# Author Correction: A new Gondwanan mayfly family from the Lower Cretaceous Crato Formation, Brazil (Ephemeroptera: Siphlonuroidea: Astraeopteridae fam. nov.)

**DOI:** 10.1038/s41598-023-46324-4

**Published:** 2023-11-06

**Authors:** Arianny P. Storari, Arnold H. Staniczek, Roman J. Godunko

**Affiliations:** 1 Laboratório de PaleontologiaDepartamento de Ciências Biológicas, Centro de Ciências Humanas e Naturais, Universidade Federal do Espírito Santo, Vitória, Brazil; 2 Department of Entomology, State Museum of Natural History Stuttgart, Stuttgart, Germany; 3 Biology Centre of the Czech Academy of Sciences, Institute of Entomology, České Budějovice, Czech Republic; 4 Department of Invertebrate Zoology and Hydrobiology, University of Łódź, Łódź, Poland

Correction to: *Scientific Reports* 10.1038/s41598-023-36778-x, published online 20 July 2023

The original version of the Article contained errors. The holotypes inventoried at the Senckenberg Gesellschaft für Naturforschung, Frankfurt/Main, Germany (SMF), have now been curated at the Museu de Paleontologia Plácido Cidade Nuvens, Santana do Cariri, Brazil (MPSC). The fossil specimens have received new inventory numbers as follows: *Astraeoptera vitrea* Storari, Staniczek & Godunko, 2023, formerly SMF VI 1026, has received inventory number MPSC I 7437; *Astraeoptera oligovenata* Storari, Staniczek & Godunko, 2023, formerly SMF VI 743, has received inventory number MPSC I 7438; *Eosophobia acuta* Storari, Staniczek & Godunko, 2023, formerly SMF VI 802, has received inventory number MPSC I 7439.

Consequently, in the legend of Figure 1,

“(b) *Astraeoptera vitrea* sp. nov., SMF VI 1026. (c) *Astraeoptera oligovenata* sp. nov., SMF VI 743. (d) *Eosophobia acuta* gen. et sp. nov., SMF VI 802.”

now reads

“(b) *Astraeoptera vitrea* sp. nov., MPSC I 7437. (c) *Astraeoptera oligovenata* sp. nov., MPSC I 7438. (d) *Eosophobia acuta* gen. et sp. nov., MPSC I 7439.”

In the legend of Figure 3,

“*Astraeoptera vitrea* sp. nov., holotype, adult male, SMF VI 1026.”

now reads

“*Astraeoptera vitrea* sp. nov., holotype, adult male, MPSC I 7437.”

In the legend of Figure 4,

“*Astraeoptera oligovenata* sp. nov., holotype, adult [?] female, SMF VI 743.”

now reads

“*Astraeoptera oligovenata* sp. nov., holotype, adult [?] female, MPSC I 7438.”

In the legend of Figure 5,

“*Eosophobia acuta* sp. nov., holotype, adult female, SMF VI 802.”

now reads

“*Eosophobia acuta* sp. nov., holotype, adult female, MPSC I 7439.”

In the legend of Figure 6,

“*Eosophobia acuta* sp. nov., holotype, adult female, SMF VI 802.”

now reads

“*Eosophobia acuta* sp. nov., holotype, adult female, MPSC I 7439.”

Additionally, in the Results section, under the subheading ‘Systematic Paleontology’,

“**Species composition.** *Astraeoptera cretacica* Brandão et al. 2021 [type species; adult female]; *Astraeoptera vitrea* sp. nov. [SMF VI 1026; adult male]; *Astraeoptera oligovenata* sp. nov. [SMF VI 743; adult of putative female].”

now reads

“**Species composition.** *Astraeoptera cretacica* Brandão et al. 2021 [type species; adult female]; *Astraeoptera vitrea* sp. nov. [MPSC I 7437; adult male]; *Astraeoptera oligovenata* sp. nov. [MPSC I 7438; adult of putative female].”

In the same section,

“**Type material. Holotype.** Adult male, inventory number SMF VI 1026 (collection of Senckenberg Naturmuseum, Frankfurt/Main, Germany).”

now reads

“**Type material. Holotype.** Adult male, inventory number MPSC I 7437 (collection of Museu de Paleontologia Plácido Cidade Nuvens, Santana do Cariri, Brazil).”

and

“**Type material. Holotype.** Adult [?] female, inventory number SMF VI 743 (collection of Senckenberg Naturmuseum, Frankfurt/Main, Germany).”

now reads

“**Type material. Holotype.** Adult [?] female, inventory number MPSC I 7438 (collection of Museu de Paleontologia Plácido Cidade Nuvens, Santana do Cariri, Brazil).”

Finally,

“**Type material. Holotype.** Adult female, inventory number SMF VI 802 (collection of the Senckenberg Naturmuseum, Frankfurt/Main, Germany).”

now reads

“**Type material. Holotype.** Adult female, inventory number MPSC I 7439 (collection of Museu de Paleontologia Plácido Cidade Nuvens, Santana do Cariri, Brazil).”

Additionally, in the Discussion an incorrect family name was previously called out and,

“Apart from Astraeoptera fam. nov. found in the Cretaceous of Brazil, only a few Gondwanan representatives of Siphlonuroidea of the Southern Hemisphere have been reported from the Cretaceous of Australia^18^.”

now reads

“Apart from Astraeopteridae fam. nov. found in the Cretaceous of Brazil, only a few Gondwanan representatives of Siphlonuroidea of the Southern Hemisphere have been reported from the Cretaceous of Australia^18^.”

Furthermore, in the Materials and methods section,

“The remaining material of this study is housed in the fossil collection of Senckenberg Naturmuseum, Frankfurt/Main, Germany [SMF], who had acquired it in 2004 from Fossils Worldwide, Sulzbachtal, Germany, and later kindly made it available to us for investigation.”

now reads

“The remaining material was studied between 2016 and 2019 as a part of the fossil collection of Senckenberg Naturmuseum, Frankfurt/Main, Germany [SMF], who had acquired it in 2004 from Fossils Worldwide, Sulzbachtal, Germany, and later kindly made it available to us for investigation. Due to an exchange of specimens under the framework of an ongoing cooperation of the Senckenberg Gesellschaft für Naturforschung, Frankfurt/Main, Germany (SMF), with the Universidade Regional do Cariri (URCA), Crato, Brazil, the following holotypes are now curated at the Museu de Paleontologia Plácido Cidade Nuvens, Santana do Cariri, Brazil (MPSC): *Astraeoptera vitrea* Storari, Staniczek & Godunko, 2023 (former inventory number SMF VI 1026) has received inventory number MPSC I 7437, the holotype of *Astraeoptera oligovenata* Storari, Staniczek & Godunko, 2023 (inventory number SMF VI 743) has received the inventory number MPSC I 7438, and the holotype of *Eosophobia acuta* Storari, Staniczek & Godunko, 2023 (inventory number SMF VI 802) has received the inventory number MPSC I 7439.”

In the Data availability section,

“All fossil specimens used in this study are housed in the public institutional collection of the Senckenberg Forschungsinstitut und Naturmuseum (Frankfurt, Germany), as specified in the Section “Material and methods”.”

now reads

“All fossil specimens newly described in this study are housed in the institutional collection of Museu de Paleontologia Plácido Cidade Nuvens, Santana do Cariri, Brazil (MPSC), as specified in the Section “Material and methods”.”

Finally, the Supplementary Table 1 also contained the previous holotype inventory numbers. This error has now been corrected in the Supplementary Information file that accompanies the original Article.

The original Article and accompanying Supplementary Information file have been corrected.

### Supplementary Information

Supplementary Table 1.

