# Supplementary material for: A new Gondwanan mayfly family from the Lower Cretaceous Crato Formation, Brazil (Ephemeroptera: Siphlonuroidea: Astraeopteridae fam. nov.)
Source: Sci Rep. 2023 Jul 20;13:11735. doi: 10.1038/s41598-023-36778-x (PMC10359310; doi:10.1038/s41598-023-36778-x)
Supplement: Supplementary file 2 — Supplementary Table 2. [file 41598_2023_36778_MOESM2_ESM.docx]

**Supplementary Table 2.** List of taxa described as fossil Siphlonuroidea.

| **Species** | **Distribution** |
| --- | --- |
| *†Albisca* Sinitshenkova 1989* | Cretaceous of Mongolia |
| *†Astraeoptera* Brandão et al. 2021 | Cretaceous of Brazil |
| *†Australurus* Jell and Duncan 1986* | Cretaceous of Australia |
| *†Baltameletus* Demoulin 1968 | Eocene of Russian Federation |
| *†Balticophlebia* Demoulin 1968* | Eocene of Russian Federation |
| *†Bolbonyx* Sinitshenkova 1990* | Jurassic of the Russian Federation |
| *†Cheirolgisca* Lin and Huang 2008 | Jurassic of China |
| *†Cretoneta* Tshernova 1971* | Cretaceous of the Russian Federation |
| *†Dulcimanna* Jell and Duncan 1986* | Cretaceous of Australia |
| *†Jurassonurus* Huang et al. 2008* | Jurassic of China |
| *†Mesobaetis* Brauer et al. 1889* | Triassic of Ukraine; Jurassic of China, Mongolia, and the Russian Federation; Cretaceous of the Russian Federation |
| *†Mogzonurella* Sinitshenkova 1985* | Jurassic of the Russian Federation |
| *†Mogzonurus* Sinitshenkova 1985* | Jurassic of Russian Federation |
| *†Multiramificans* Huang et al. 2007 | Jurassic of China |
| *†Olgisca* Demoulin 1970* | Jurassic of China and Germany |
| *†Proameletus* Sinitshenkova 1976* | Jurassic and Cretaceous of the Russian Federation |
| *†Promiara* Jell and Duncan 1986 | Cretaceous of Australia |
| *†Siphangarus* Sinitshenkova 2000* | Cretaceous of the Russian Federation |
| *Siphlonurus* Eaton 1868 | Eocene of the Russian Federation to present |
| *†Stackelbergisca* Tshernova 1967 | Cretaceous of the Russian Federation |
| *†Triassonurus* Sinitshenkova and Marchal-Papier 2005* | Triassic of France |
| *†Xenophlebia* Demoulin 1968 | Eocene of Russian Federation |

**incertae sedis* according to N.J. Kluge [11].
