# Supplementary material for: Author Correction: A new Gondwanan mayfly family from the Lower Cretaceous Crato Formation, Brazil (Ephemeroptera: Siphlonuroidea: Astraeopteridae fam. nov.)
Source: Sci Rep. 2023 Nov 6;13:19274. doi: 10.1038/s41598-023-46324-4 (PMC10630417; doi:10.1038/s41598-023-46324-4)
Supplement: Supplementary file 1 — Supplementary Table 1. [file 41598_2023_46324_MOESM1_ESM.docx]

**Supplementary Table 1.** Character list of adult *Astraeoptera* (Crato Formation, Lower Cretaceous, Brazil).

| **Characters** | ***Astraeoptera cretacica* Brandão *et al*., 2021**  **HOLOTYPE** | ***Astraeoptera vitrea* sp. nov.**  **SMF VI1026**  **HOLOTYPE** | ***Astraeoptera oligovenata* sp. nov.**  **SMF VI743**  **HOLOTYPE** | ***Eosophobia acuta* sp. nov.**  **SMF VI802**  **HOLOTYPE** |
| --- | --- | --- | --- | --- |
| ***Sex*** | ***Female*** | ***Male*** | ***? Female*** | ***Female*** |
|  |  |  |  |  |
| **Measurements** |  |  |  |  |
| Body length [mm] | 8.00* | 10.40* | 13.20* | 7.60* |
| Forewings length [mm] | 7.65* | 8.45* | 11.40 | 7.70* |
| Forewing width [mm] | 3.62* | 4.53* | 6.30 | 3.70* |
| Forewing [width/length ratio] | 0.47 | 0.54 | 0.55 | 0.48 |
| Hind wings length [mm] | 1.93* | 3.72* | al least 2.0* | 3.40* |
| Hind/forewing length ratio | 0.25 | 0.44 | (?) 0.18 | 0.44 |
| Terminal filaments [mm] | 6.70* | 3.00* | – | – |
|  |  |  |  |  |
| **Thorax** |  |  |  |  |
| Prothorax length [mm] | 0.42* | 0.38* | 0.80* | 0.47* |
| Pterothorax length [mm] | 2.40* | 3.38* | 5.00* | 3.50* |
| Pterothorax/body length ratio | 0.30 | 0.33 | 0.38 | 0.46 |
| Pterothorax/abdomen length ratio | 0.48 | 0.50 | 0.54 | 0.82 |
|  |  |  |  |  |
| ***Mesothorax*** |  |  |  |  |
| *Mesonotal suture* | stretched backward medially | – | (?) stretched backward medially | stretched backward medially |
| *Lateroparapsidal suture* | concave centrally,  bent inward distally | – | elongated,  bent inward distally | concave centrally,  bent inward distally |
| *Anterior paracoxal suture* | elongated, (?) complete | (?) elongated, (?) complete | – | elongated, complete |
| *Basisternum of mesothorax* | relatively large | (?) relatively large | relatively short | not large |
| *Furcasternal protuberances* | relatively large | (?) relatively large | – | relatively large,  tapers anteriorly |
|  |  |  |  |  |
| **Forewing** |  |  |  |  |
| Costal brace | (?) arched, (?) elongated | arched, slightly pronounced | – | strongly arched |
| C–Sc area [shape] | not widened centrally | not widened centrally | Wide centrally | narrow centrally |
| C–Sc area [cross venation] | 11 simple veins* | 17 simple veins*  + 5 forked veins | 6 simple veins* | 13 simple veins*  + (?) 5 forked veins |
| *Pterostigma [shape of veins]* | forked | – | – | simple/forked |
| *Pterostigma [number of veins]* | ? 3[4]* | – | – | ? 5* |
| Sc–RA area [shape] | tapered centrally,  narrower than C–Sc | tapered centrally,  narrower than C–Sc | tapered distally,  slightly wider C–Sc | tapered centrally,  narrower than C–Sc |
| Sc–RA area [cross venation] | 17 simple veins* | 13 simple veins* | – | at least 8* |
| RP fork [place of furcation] | 0.15 | 0.23 | 0.15 | 0.22 |
| RP2 fork [place of furcation] | 0.60 | 0.48 | 0.26 | 0.20 |
| MA fork | slightly asymmetrical | nearly symmetrical | nearly symmetrical | nearly symmetrical |
| MA fork [place of furcation] | 0.60 | 0.46 | 0.58 | 0.65 |
| MA1 [shape] | bent | moderately bent | moderately bent centrally  and basally | moderately bent |
| MA2 [shape] | nearly straight | nearly straight | slightly bent centrally | moderately bent |
| MP fork | slightly asymmetrical | (?) asymmetrical | – | (?) asymmetrical |
| MP fork [place of furcation] | 0.13 | – | – | 0.07 |
| MP1–iMP + iMP – MP2 areas  [number of elongated intercalaries] | 2+2 | 0+1 | 1+0 | 2+1 |
| MP2–CuA area  [number of elongated intercalaries] | 2 | 1+one short | 1+one short | 0+one short |
| MP2/CuA relations | sub-parallel proximally,  convergent distally | convergent distally | convergent distally | sub-parallel proximally,  slightly convergent distally |
| Cubital field [shape] | relatively narrow | relatively narrow | relatively narrow | relatively narrow |
| Cubital field  [overall number of veins] | 8* | 7* | 3* | 7* |
| Cubital field  [number of elongated veins nearly sub-parallel to CuA] | 2* | 2* | – | 0 |
| Cubital field  [number of elongated veins nearly sub-parallel to CuP] | 2* | 2* | – | 2* |
| Cubital field  [cross veins between CuA, CuP and intercalary veins] | occasional 3 veins*,  poorly preserved | occasional 3[?4] veins between CuA and iCu1 | not recognizable | not recognizable |
| CuP–A1 area  [number of intercalary veins] | – | – | – | 1 |
| CuP–A1 area  [number of cross veins] | not recognizable | – | 1+1 [shortened] | 1 [indistinct] |
| Anal venation | poorly preserved,  one visible vein | poorly preserved,  at least 2 veins | poorly preserved | poorly preserved,  at least 2 veins |
| A1 [shape] | strongly bent distally | moderately bent centrally | strongly bent distally | moderately bent centrally |
|  |  |  |  |  |
| **Abdomen** |  |  |  |  |
| Largest segments | VII–VIII | VII | poorly preserved | V–VI |
| Subgenital plate [shape] | large, expanded proximally | – | – | – |

* – as preserved.
